# Individual and community predictors of urinary ceftriaxone-resistant *Escherichia coli* isolates, Victoria, Australia

**DOI:** 10.1186/s13756-019-0492-8

**Published:** 2019-02-12

**Authors:** Kyra Y. L. Chua, Andrew J. Stewardson

**Affiliations:** 1 Department of Microbiology, Dorevitch Pathology, Heidelberg, Victoria Australia; 2 Department of Infectious Diseases and Department of Microbiology, Austin Health, Heidelberg, Victoria Australia; 3 Department of Infectious Diseases, Alfred Hospital and Central Clinical School, Monash University, Melbourne, Victoria Australia

**Keywords:** (MeSH), *Escherichia coli*, Drug resistance, bacterial, Ceftriaxone, Urinary tract infections, Demography, Censuses, Australia, Victoria

## Abstract

**Background:**

Ceftriaxone-resistant Enterobacteriaceae are priority pathogens of critical importance. *Escherichia coli* is the most commonly isolated Enterobacteriaceae. There are few data regarding non-invasive ceftriaxone-resistant *E. coli* (CR-EC) isolates in the Australian community. We aimed to describe the prevalence, phenotype, geographic variation, and sociodemographic predictors of ceftriaxone-resistance among *E. coli* isolates recovered from urine specimens.

**Methods:**

In August 2017, we prospectively analysed *E. coli* isolates recovered from urine specimens submitted to Dorevitch Pathology (Victoria, Australia), a laboratory that services patients in the community and hospitals. In addition to patient-level predictors of ceftriaxone resistance, we mapped patient postcodes to community-level indicators including Index of Relative Socioeconomic Deprivation, remoteness, and proportion of residents born overseas. We used Poisson regression with log link and robust standard errors to quantify the association between ceftriaxone resistance and patient- and community-level factors.

**Results:**

We included 6732 non-duplicate *E. coli* isolates. Most (89.2%, 6008/6732) were obtained from female patients. Median age was 56 years (IQR, 32–74). Most patients (90.5%, 5789/6732) were neither referred from a hospital nor residing in a residential aged care facility (RACF). Among the 6732 isolates, 5.7% (382) were CR-EC, ranging from 3.5% (44/1268) in inner regional areas to 6.3% (330/5267) in major cities. Extended spectrum ß–lactamase (ESBL) -production was the most common mechanism for ceftriaxone resistance (89%, 341/382). Nitrofurantoin was the most active oral agent against CR-EC. Eight CR-EC isolates (2.4%) were susceptible only to amikacin, meropenem and nitrofurantoin. None were resistant to meropenem. On multivariable analysis, ceftriaxone resistance was associated with age, residence in a RACF (adjusted relative risk [aRR] 2.94, 95% confidence interval [CI] 2.10–4.13), specimen referral from hospital (aRR 2.05, 95% CI 1.45–2.9), and the proportion of residents born in North Africa and the Middle East (aRR 1.30 for each 5% absolute increase, 95% CI 1.09–1.54), South-East Asia (aRR 1.14, 95% CI 1.02–1.27), and Southern and Central Asia (aRR 1.16, 95% CI 1.04–1.28).

**Conclusions:**

These results provide insights into sociodemographic variation in CR-EC in the community. A better understanding of this variation may inform empiric treatment guidelines and strategies to reduce community dissemination of CR-EC.

## Background

Ceftriaxone is a third-generation cephalosporin antibiotic frequently used to treat invasive infections caused by Enterobacteriaceae such as *Escherichia coli*. The globally increasing prevalence of antimicrobial resistance (AMR) among Enterobacteriaceae is resulting in increased patient morbidity and mortality, increased healthcare costs, and increased use of last-line antibiotics [1, 2]. The World Health Organization has therefore recently recognized ceftriaxone-resistant Enterobacteriaceae as priority pathogens of critical importance [3]. The most common mechanisms of ceftriaxone resistance among Enterobacteriaceae are extended-spectrum ß-lactamases (ESBLs) and AmpC ß–lactamases. The genes encoding these enzymes are horizontally transmissible between bacteria, facilitating the spread of resistance [1].

Although the hospital setting has traditionally been considered a reservoir for the amplification of drug-resistant organisms, recent evidence suggests that there is likely to be substantial dissemination of antimicrobial resistance in the community [4]. There is emerging literature pointing to the potential importance of community-level sociodemographic predictors of antimicrobial resistance [5, 6]. While Australia has a hospital-based national surveillance system for antimicrobial susceptibility in invasive Enterobacteriaceae isolates, the prevalence and predictors of ceftriaxone-resistant *E. coli* (CR-EC) in the community are less well described [7].

We aimed to describe the prevalence of ceftriaxone-resistance among *E. coli* isolates recovered from urine specimens and determine individual- and community-level predictors of ceftriaxone resistance among these isolates.

## Methods

### Setting

Victoria is the second-most populous state in Australia, with an estimated 6.2 million residents, 28.3% of whom are born overseas [8]. The top five countries of birth other than Australia are England (2.9%), India (2.9%), China (2.7%), New Zealand (1.6%) and Vietnam (1.4%) [8]. Dorevitch Pathology, Heidelberg (Primary Health Care, Victoria, Australia) is a commercial laboratory that services metropolitan Melbourne (the state capital) and regional Victoria and receives approximately 50,000 urine samples monthly for microscopy and culture. Most specimens are referred by general practitioners from patients in the community, with fewer samples from public and private hospitals.

### Bacterial isolates

*E. coli* isolates from urine samples submitted to Dorevitch Laboratory, Heidelberg, in August 2017 were examined. Only the first urine isolate from each patient was included. *E. coli* isolates were identified using chromID CPS agar (bioMérieux, Marcy-l’Étoile, France) according to the manufacturer’s instructions with isolates typically displaying pink to burgundy colonies and a positive reaction using spot indole reagent (Remel, San Diego, USA). The identity of ceftriaxone resistant *E. coli* was confirmed using matrix-assisted laser desorption ionization-time of flight mass spectrometry on the VITEK MS platform (bioMérieux).

### Phenotypic characterization of isolates

Direct disc susceptibility testing was routinely performed for urine isolates using a panel of agents including ceftriaxone. Isolates found to be ceftriaxone not susceptible (i.e. ceftriaxone intermediate or resistant, with zones of inhibition < 23 mm) were tested using VITEK2 (AST-N247 cards, bioMérieux) to confirm ceftriaxone resistance and to determine susceptibility to other antibiotics. Categorical interpretation of the VITEK2 MICs were performed using the 2017 CLSI guidelines [9].

Phenotypic testing of ceftriaxone resistant isolates for the presence of ESBL or AmpC ß-lactamases was performed using the modified CLSI method as previously described [10]. Briefly, a lawn culture of the test isolate was prepared onto which were placed, 30 μg cefotaxime and 30 μg ceftazidime discs (BD BBL Sensi-Disc, Becton Dickinson, Franklin Lakes, USA) with and without 10 μg clavulanate, and with and without the addition of 400 μg phenylboronic acid and 292 μg EDTA and incubated for 18 h in air at 35 °C. An augmentation of ≥5 mm in the inhibition zone diameter of the clavulanic acid containing discs was considered a positive result for ESBL production. Where the modified CLSI method suggested the presence of an AmpC ß–lactamase, this was confirmed using the AmpC disk test as previously described [11]. Briefly the test organism was inoculated onto a paper disc impregnated with Tris-EDTA and placed in close proximity with a cefoxitin 30 μg disc onto a lawn culture of *E. coli* ATCC 25922 and incubated overnight in air at 35 °C. The expression of an AmpC ß–lactamase was demonstrated by an indentation or flattening of the zone of inhibition. Ceftriaxone resistant isolates were classified into those expressing ESBL ß–lactamases, AmpC ß–lactamases or both ESBL and AmpC ß–lactamases.

Isolates with a meropenem MIC of > 0.25 μg/mL by VITEK2 were tested by the modified carbapenem inactivation method as described in the 2017 CLSI guidelines [9]. These were also submitted to the reference laboratory, Microbiological Diagnostic Unit Public Health Laboratory (Melbourne, Australia), for genotypic detection of the presence of carbapenemase ß–lactamase gene targets including GES, IMI, KPC, SME, IMP, NDM, VIM, OXA-23-like, OXA-48-like, OXA-51-like, and OXA-58-like.

### Data sources

Demographic data associated with each isolate was collected by interrogating the laboratory information system for patient age (in years on date of sample collection), sex, postcode of patient residence, referral source (community or hospital), and patient residence in a residential aged care facility (RACF). We mapped each postcode to its corresponding Victorian electoral region, Australian Statistical Geography Standard areas (Statistical Area Level 4 and Remoteness Area category), and decile of the 2016 Index of Relative Socio-economic Disadvantage (IRSD) using resources from the Victorian Electoral Commission and Australian Bureau of Statistics [12–15]. Remoteness areas divide Australia into five categories on the basis of relative access to services; Major Cities, Inner Regional, Outer Regional, Remote, and Very Remote [13]. The IRSD is a socio-economic index that measures disadvantage by summarizing “a range of information about the economic and social conditions of people and households within an area” [14]. We used postcode-level data on country of birth from the 2016 Australian census to compute the proportion of persons in each postcode born in each of the nine ‘major groups’ of countries defined by the Standard Australian Classification of Countries [16, 17].

### Statistical analysis

We described continuous variables using median with interquartile range (IQR) and categorical variables using count and percentage with binomial 95% confidence intervals (CIs). Comparisons of categorical variables between two or more groups were performed using Fisher exact test. We quantified the association between ceftriaxone resistance and various predictors using Poisson regression models with log link and robust standard errors to compute risk ratios with 95% confidence intervals [18]. This analysis was performed at the level of individual *E. coli* isolates. We considered a set of patient-level predictors (sex, age, referral source and residence in a RACF) and postcode-level predictors (region, 1st IRSD decile, and proportion of residents born overseas). We included proportion of residents born overseas as a continuous variable, but divided the value (expressed as a percentage) by five so that the risk ratio could be interpreted as the change in CR-EC associated with each absolute 5% change in percentage of residents born in the region in question. We forced patient-level predictors into the multivariable model and initially included all postcode-level predictors. We then removed any postcode-level predictors from the base model where this resulted in a significant reduction in the Akaike Information Criteria according to the likelihood ratio test. We compared the final model with a mixed-effects Poisson regression model including the same fixed effects, but with the addition of postcode as a random effect. Given only minor differences in results, we have reported the simpler model (without a random effect). All statistical analyses were performed with R, version 3.5.1 (R Foundation for Statistical Computing, Vienna, Austria), including ‘tidyverse’ and ‘sandwich’ packages [19, 20].

## Results

### Description of cohort

There were 6732 non-duplicate *E. coli* isolates recovered from urine specimens in August 2017. Most (89.2%, 6008/6732) isolates were obtained from female patients. Patients ranged in age from 1 week to 102 years, with a median of 56 years (interquartile range [IQR, 32–74]). Age was missing for 3 patients. There were 416 (6.2%) isolates from patients aged 12 years or less.

There were 637 (9.5%) isolates referred from a hospital. Of these, 56.2% (358/637) were referred from patients in the emergency department, with the remaining 43.8% (279/637) isolates from other hospital wards. There were 335 (5.0%) isolates from patients who lived in a RACF. Most isolates (90.5%, 5789/6732) were from patients neither referred by a hospital nor residing in an RACF.

Patient residential address postcodes were available for 6726 isolates (99.9%). Patients lived in 598 distinct postcodes, with a median (IQR) of 5 (2–14) isolates per postcode. Ninety-nine and 196 postcodes were represented by least 20 and at least 10 isolates, respectively. Most patients lived in a ‘Major City’ (68.1% [5322/6732]) or ‘Inner Regional’ area (24.8% [1937/6732]), with 186, 1, and 4 patients from ‘Outer Regional’, ‘Remote’, and ‘Very Remote’ areas, respectively.

### Microbiology of ceftriaxone-resistant *E. coli* isolates

The overall prevalence of ceftriaxone resistance was 5.7% (382/6732). Using phenotypic characterization, we found that ESBL-production was the most common mechanism of ceftriaxone resistance: 87.4% (334/382) of CR-EC isolates expressed only ESBL enzymes, 10.7% (41/382) expressed only AmpC ß-lactamases, and 1.8% (7/382) expressed both.

**Fig. 1:**
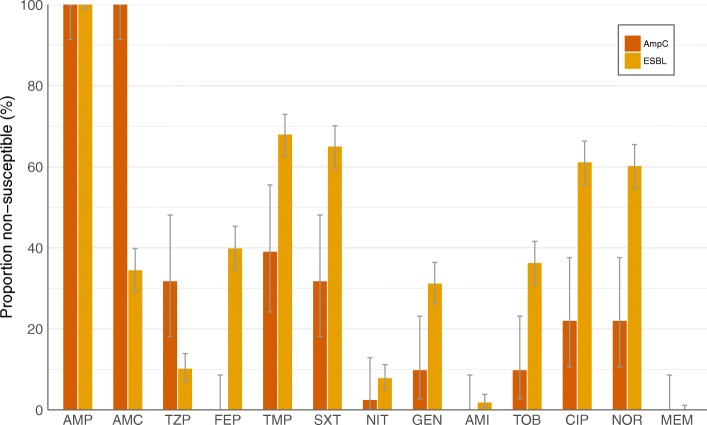
Susceptibility of ceftriaxone-resistant *Escherichia coli*, by phenotype. Includes data from 41 AmpC isolates and 334 ESBL-producing isolates. Seven isolates expressing ESBL plus AmpC have been omitted. AMC = amoxicillin–clavulanate; AMI = amikacin, AMP = ampicillin; AmpC = AmpC ß-lactamases; CIP = ciprofloxacin; ESBL = extended-spectrum ß-lactamases; FEP = cefepime; GEN = gentamicin; MEM = meropenem; NIT = nitrofurantoin; NOR = norfloxacin; TOB = tobramycin; TZP = piperacillin–tazobactam; SXT = trimethoprim–sulfamethoxazole; TMP = trimethoprim

Phenotypic susceptibility to other antibiotics are demonstrated in Fig. 1. Meropenem was the most active agent. Two isolates demonstrated a VITEK2 meropenem MIC of > 0.25 μg/mL. One of these had an ESBL phenotype and the other had an AmpC phenotype. Both of these had a meropenem MIC of 0.5 μg/mL and testing by modified carbapenem inactivation method did not demonstrate any phenotypic carbapenemase activity. No carbapenemase genes were detected in either isolate. Hence all isolates were meropenem susceptible.

Most isolates also retained susceptibility to amikacin with only four isolates (1.0%) demonstrating intermediate susceptibility and 2 isolates (0.5%) demonstrating resistance.

Nitrofurantoin was the most active orally available antibiotic for CR-EC. There were four ESBL-only isolates (1.2%) that were resistant and 22 (6.6%) that were intermediate susceptibility; no AmpC-only isolates that were resistant and 1 (2.4%) that had intermediate susceptibility.

In ESBL-only isolates, amoxicillin/clavulanate also retained modest activity. Ciprofloxacin, trimethoprim and trimethoprim/sulfamethoxazole were more active against AmpC-only isolates (Fig. 1).

Overall, the AmpC isolates were more susceptible than the ESBL isolates to the non ß-Lactam antibiotics. There were 8 isolates (2.4%), all of which were ESBL-only isolates, that were only susceptible to amikacin, meropenem and nitrofurantoin.

### Geographic distribution of CR-EC

**Fig. 2:**
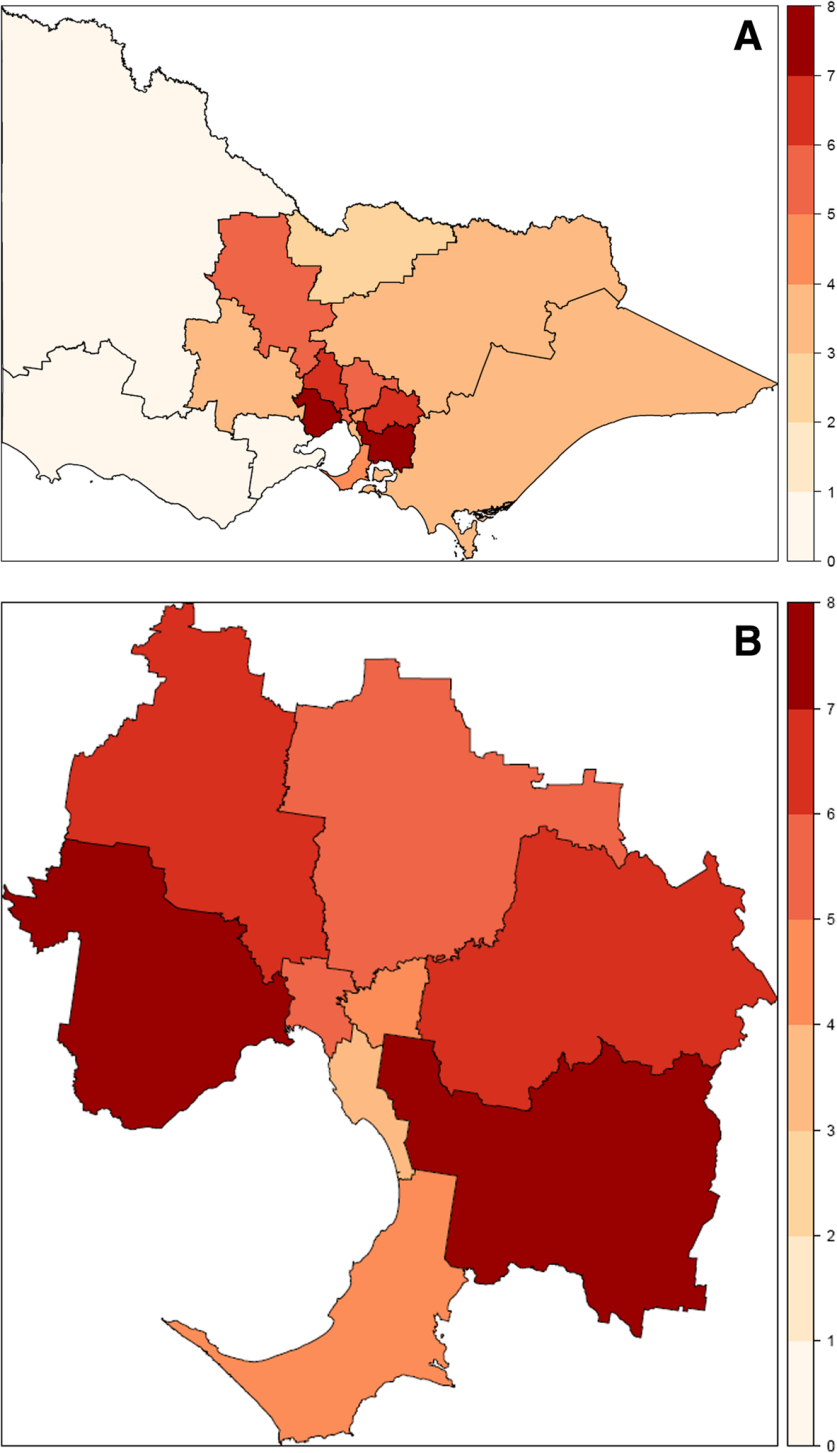
Geographic distribution of ceftriaxone resistance among urinary *Escherichia coli* isolates in Victoria (**a**) and the Greater Melbourne area (b). Scale represents percentage of *E. coli* isolates that are resistant to ceftriaxone. 943 isolates from patients living in a RACF and/or referred from a hospital have been omitted. Geographic boundaries correspond to Statistical Area Level 4 defined by the Australian Statistical Geography Standard

We excluded 943 isolates from patients living in a RACF and/or referred from a hospital to describe geographic variation in CR-EC prevalence in the community (Fig. 2). Overall CR-EC prevalence in this group was 5.0% (291/5789). This was higher in Major Cities (5.6%, 252/4507) than Inner Regional (3.1%, 34/1109) or more remote areas (3.0%, 5/167). Within metropolitan Melbourne, there is geographic variation in the prevalence of CR-EC (Fig. 2b).

### Factors associated with ceftriaxone resistance

**Fig. 3:**
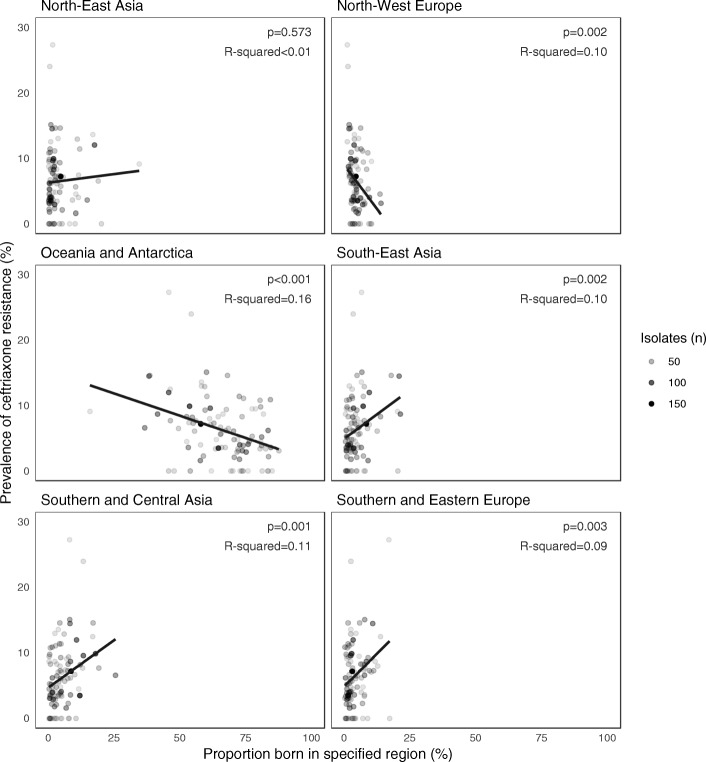
Association between prevalence of ceftriaxone resistance among urinary *Escherichia coli* isolates and region of birth of residents. Each point represents aggregate data from one postcode. Intensity of the point represents number of *E. coli* isolates represented (postcodes with less than 20 isolates have been omitted). Line of best fit computed using linear regression to predict prevalence of ceftriaxone resistance from proportion of residents born in the relevant geographic region, weighted by number of isolates. The *p*-values test the null hypothesis that the line of best fit has a coefficient (slope) of zero. R^2^ values represent the proportion of variation between postcodes in prevalence of ceftriaxone resistance that is explained by the model

Participant characteristics stratified by ceftriaxone-susceptibility is presented in Table 1. Residence in a RACF was more common among patients with CR-EC than those with ceftriaxone susceptible *E. coli,* CS-EC (11.8% [45/382] vs. 4.6% [290/6350], *P* < 0.001). Likewise, specimen referral from a hospital setting (emergency department, ED and non-ED combined) was more common among patients with CR-EC than those with CS-EC (13.1% [50/382] vs 9.2% [587/6350], *P* = 0.015). When aggregated at postcode level, there was an association between prevalence of ceftriaxone resistance and country of birth (Fig. 3).

**Table 1:** Individual and community-level characteristics of patients with ceftriaxone-susceptible and resistant urinary *Escherichia coli* isolates

Characteristic	*Escherichia coli* isolates	
	Ceftriaxone susceptible	Ceftriaxone resistant
	*N = 6350*	*N = 382*
*Patient-level characteristics*		
Sex		
Female	5677 (89.4%)	331 (86.6%)
Male	673 (10.6%)	51 (13.4%)
Age group		
Median (IQR)	56 (32–74)	58 (31–75)
Age 12 or less	394 (6.2%)	22 (5.8%)
Not available	3 (0%)	0
Residence in RACF		
No	6060 (95.4%)	337 (88.2%)
Yes	290 (4.6%)	45 (11.8%)
Referral source		
Community	5763 (90.8%)	332 (86.9%)
Hospital: ED	343 (5.4%)	15 (3.9%)
Hospital: non-ED	244 (3.8%)	35 (9.2%)
*Postcode-level characteristics*		
Region		
Northern Metropolitan	613 (9.7%)	59 (15.4%)
Eastern Metropolitan	663 (10.4%)	47 (12.3%)
South-Eastern Metropolitan	911 (14.3%)	70 (18.3%)
Southern Metropolitan	632 (10%)	28 (7.3%)
Western Metropolitan	904 (14.2%)	78 (20.4%)
Northern Victoria	735 (11.6%)	28 (7.3%)
Eastern Victoria	922 (14.5%)	51 (13.4%)
Western Victoria	264 (4.2%)	6 (1.6%)
Other	699 (11%)	15 (3.9%)
Not available	7 (0.1%)	0
Remoteness area		
Major Cities	4989 (67.5%)	333 (78.4%)
Inner Regional	1865 (25.2%)	72 (16.9%)
Outer Regional	509 (6.9%)	20 (4.7%)
Remote	26 (0.4%)	0
Very Remote	4 (0.1%)	0
Index of Relative Socio-economic Disadvantage, decile^a^		
1	542 (8.5%)	49 (12.8%)
2	345 (5.4%)	11 (2.9%)
3	448 (7.1%)	24 (6.3%)
4	582 (9.2%)	33 (8.6%)
5	613 (9.7%)	36 (9.4%)
6	746 (11.7%)	58 (15.2%)
7	695 (10.9%)	42 (11%)
8	846 (13.3%)	44 (11.5%)
9	945 (14.9%)	52 (13.6%)
10	568 (8.9%)	32 (8.4%)
Not available	20 (0.3%)	1 (0.3%)
Percentage of postcode residents born in region, median (IQR)		
Oceania and Antarctica	70.1 (58.0–78.3)	62.9 (53.7–74.4)
North-West Europe	4.5 (3.2–6.6)	4.0 (2.4–6.0)
Southern and Eastern Europe	2.4 (1.2–4.3)	2.8 (1.6–5.8)
North Africa and the Middle East	0.7 (0.2–1.3)	1.0 (0.5–1.7)
South-East Asia	2.7 (1.0–5.3)	3.6 (1.7–7.1)
North-East Asia	1.5 (0.5–3.9)	1.8 (0.7–4.4)
Southern and Central Asia	2.9 (0.8–7.1)	4.6 (1.5–8.6)
Americas	0.5 (0.4–0.7)	0.5 (0.4–0.7)
Sub-Saharan Africa	0.6 (0.3–1.0)	0.6 (0.4–1.0)
Not available	9.3 (7.4–11.0)	9.6 (7.7–11.6)

*ED*, Emergency department; *IQR*, interquartile range; *RACF*, residential aged-care facility. ^a^The 1st decile represents the most disadvantaged group

Results from the univariable and multivariable Poisson regression models are presented in Table 2. Of the patient-level variables, age 20–29 years, referral from a hospital, and residence in a RACF were associated with increased risk of ceftriaxone resistance on univariable regression, but patient sex was not. Among the postcode-level predictors, risk of resistance appeared to be inversely associated with remoteness, both according to the Remoteness Area classification and electoral region. Residence in a postcode belonging to the first decile of the IRSD (i.e. the most disadvantaged postcodes) was associated with increased risk of ceftriaxone resistance. Finally, the proportion of residents born in the following regions was associated with an increased risk of ceftriaxone resistance; Southern and Eastern Europe, North Africa and the Middle East, South-East Asia, North-East Asia, Southern and Central Asia, and Sub-Saharan Africa.

**Table 2:** Risk factors for ceftriaxone resistance among urinary *Escherichia coli* isolates

Characteristic	Proportion ceftriaxone resistant	Risk ratio (95% CI)^*^	Adjusted risk ratio (95% CI)^†^
Sex			
Female	5.5% (331/6008)	reference	reference
Male	7% (51/724)	1.28 (0.96–1.70)	1.17 (0.88–1.55)
Age group			
80 years and more	6.4% (72/1119)	reference	reference
70–79 years	6.3% (66/1048)	0.98 (0.71–1.35)	1.22 (0.87–1.70)
60–69 years	5.4% (48/890)	0.84 (0.59–1.19)	1.12 (0.77–1.63)
50–59 years	5.6% (42/751)	0.87 (0.60–1.26)	1.20 (0.81–1.78)
40–49 years	3.9% (26/659)	0.61 (0.40–0.95)	0.84 (0.53–1.34)
30–39 years	5.3% (39/734)	0.83 (0.57–1.21)	1.05 (0.70–1.59)
20–29 years	6.9% (58/846)	1.07 (0.76–1.49)	1.48 (1.02–2.15)
15–19 years	3.3% (8/246)	0.51 (0.25–1.04)	0.77 (0.37–1.61)
10–14 years	2.8% (2/72)	0.43 (0.11–1.72)	0.66 (0.17–2.61)
5–9 years	4.1% (8/194)	0.64 (0.31–1.31)	0.98 (0.47–2.02)
0–4 years	7.6% (13/170)	1.19 (0.67–2.10)	1.71 (0.95–3.07)
Residence in RACF			
No	5.3% (337/6397)	reference	reference
Yes	13.4% (45/335)	2.55 (1.91–3.41)	2.94 (2.10–4.13)
Referral source			
Community	5.4% (332/6095)	reference	reference
Hospital: ED	4.2% (15/358)	0.77 (0.46–1.28)	0.72 (0.43–1.21)
Hospital: non-ED	12.5% (35/279)	2.30 (1.66–3.19)	2.05 (1.45–2.9)
Region			
Western Metropolitan	7.9% (78/982)	reference	reference
Northern Metropolitan	8.8% (59/672)	1.11 (0.80–1.53)	
Eastern Metropolitan	6.6% (47/710)	0.83 (0.59–1.18)	
South-Eastern Metropolitan	7.1% (70/981)	0.90 (0.66–1.23)	
Southern Metropolitan	4.2% (28/660)	0.53 (0.35–0.81)	
Northern Victoria	3.7% (28/763)	0.46 (0.30–0.70)	
Eastern Victoria	5.2% (51/973)	0.66 (0.47–0.93)	
Western Victoria	2.2% (6/270)	0.28 (0.12–0.63)	
Other	2.1% (15/714)	0.26 (0.15–0.46)	
Remoteness area			
Major Cities	6.3% (330/5267)	reference	
Inner Regional	3.5% (44/1268)	0.55 (0.41–0.75)	0.89 (0.60–1.30)
Other	4.2% (8/191)	0.67 (0.34–1.33)	1.13 (0.56–2.31)
Index of Relative Socio-economic Disadvantage, 1st decile			
No	5.4% (332/6120)	reference	
Yes	8.3% (49/591)	1.53 (1.15–2.04)	0.93 (0.65–1.31)
Percentage of postcode residents born in region, median (IQR)^‡^			
Oceania and Antarctica	NA	0.89 0.86–0.92)	
North-West Europe	NA	0.62 (0.49–0.77)	
Southern and Eastern Europe	NA	1.39 (1.23–1.57)	1.12 (0.94–1.32)
North Africa and the Middle East	NA	1.47 (1.31–1.65)	1.30 (1.09–1.54)
South-East Asia	NA	1.24 (1.16–1.33)	1.14 (1.02–1.27)
North-East Asia	NA	1.09 (1.01–1.19)	0.99 (0.89–1.11)
Southern and Central Asia	NA	1.29 (1.20–1.38)	1.16 (1.04–1.28)
Americas	NA	1.76 (0.49–6.33)	
Sub-Saharan Africa	NA	2.02 (1.02–3.99)	1.55 (0.66–3.65)

Notes: *From univariable Poisson regression models; †From multivariable Poisson regression model (23 records omitted due to missing values); ‡ Effect measure corresponds to the change in prevalence of ceftriaxone resistance for each 5% increase in proportion of residents born in specified region. ED = emergency department; IQR = interquartile range; RACF = residential aged-care facility

On multivariable regression, the same patient-level predictors remained significantly associated with ceftriaxone resistance, however the only postcode-level predictors to be retained in the model and significantly associated with risk of ceftriaxone resistance were the proportion of residents born in North Africa and the Middle East, South-East Asia, and Central and Southern Asia (Table 2).

## Discussion

The prevalence of ceftriaxone resistance among urinary *E. coli* isolates in Victoria was 5.7% (382/6732), with ESBL-production being the most common mechanism by phenotypic characterization. On multivariable regression, the patient-level predictors of ceftriaxone resistance were age, residence in a RACF, and referral from a hospital ward. At the community level, the proportion of residents in the patient’s postal area who were born in North Africa and the Middle East, Southern and Central Asia, and South-East Asia, was positively associated with the risk of ceftriaxone resistance. Remoteness and relative socio-economic disadvantage were associated with ceftriaxone resistance on univariable but not multivariable regression.

There are several potential explanations for the observed association between ceftriaxone resistance and the proportion of residents in a patient’s postal area that were born in North Africa and the Middle East, Southern and Central Asia, and South-East Asia. We hypothesized that this association would exist because; i) ceftriaxone resistance is likely to be more prevalent in these regions that in Australia [7, 21, 22], ii) individuals may travel to their country of birth to visit friends or relatives (VFR) or have close contact with others who do; iii) returned travellers visiting these geographic regions are at risk of colonization, and subsequent infection, with CR-EC, and iv) VFR-travellers have increased risk of travel-related infectious disease acquisition compared to travel for other reasons [23]. Alternatively, country of birth may predict other individual level risk factors for antibiotic resistance unrelated to travel that we are unable to measure, such as antibiotic consumption, household size, or diet. Importantly, we emphasize that country of birth may not be causally associated with CR-EC risk at all, but instead be a surrogate marker of other sociodemographic predictors that apply to all residents of specific postal areas.

We did not have an a priori expectation that there would be a higher prevalence of ceftriaxone resistance among isolates from individuals aged 20–29 years. We are therefore cautious in our interpretation; this result should be considered hypothesis-generating and may indeed represent a ‘chance’ finding.

Previous studies have recognized the importance of RACFs as reservoirs for antimicrobial resistance [24, 25]. This study is novel in comparing prevalence of resistance in community and RACFs from one dataset. The higher frequency of AMR in RACFs is likely multifactorial, relating to comorbidities, antibiotic exposure, and challenges in infection prevention. The prevalence of antibiotic exposure (excluding topical agents) in 292 Australian RACFs in 2017 was 6.7%, with 55% of these antibiotics prescribed without signs or symptoms of infection [26]. Urinary tract infections were the single most common indication of antibiotic therapy in four Melbourne RACFs from 2006 to 2010, with 49% (141/288) of cases of suspected UTI not fulfilling the McGeer criteria for clinical infection [27].

Our findings could be compared to those of the Australian Group on Antimicrobial Resistance (AGAR), which conducts annual hospital-based surveillance studies on bloodstream infections (BSI) caused by *Enterobacteriaceae*. The 2016 survey found that 11.8% (483/4096) of *E. coli* isolates causing BSI were not-susceptible to ceftriaxone using CLSI interpretive guidelines [28]. The higher prevalence of resistance in this estimate is likely to reflect their focus on invasive blood stream isolates in the hospital setting. Our data complement AGAR’s because non-sterile site specimens (such as urine) that reflect gut colonization may serve as an earlier indicator of dissemination of resistance in the community.

The current Australian guideline for treatment of acute cystitis in non-pregnant women recommends trimethoprim (first-line), cephalexin (second-line), and amoxicillin-clavulanate and nitrofurantoin (both third-line) [29]. As urine samples are not routinely collected for women without risk factors for drug-resistant infection, we are unable to comment on the activity of these agents for empiric treatment in general. We can, however, conclude that nitrofurantoin has the best activity against ceftriaxone-resistant *E. coli* (93% susceptible, 355/382), compared to 35% (132/382) and 57% (219/382) for trimethoprim and amoxicillin-clavulanate, respectively. This is one argument in favour of elevating nitrofurantoin to first-line therapy in line with European and USA recommendations [30].

This study has several limitations. First, while this was a prospective study (to permit extended characterization of *E. coli* isolates), we were limited to analysis of routinely collected patient data i.e. sex, age, location. In particular, we are unable to report on prior patient exposure to antibiotics or overseas travel. Second, our exploration of community-level predictors of antibiotic resistance (i.e. region, remoteness, IRSD, county of birth) are vulnerable to the ecologic bias, where associations measured at group-level may not reflect individual-level association [31]. One strength, however, of an ecologic approach is that group-level measurement of exposures may better capture their ‘complete’ effect than individual-level data given the transmissibility of ceftriaxone-resistant *E. coli* [32, 33]. Third, this one-month study cannot describe seasonal trends. Fourth, we have not performed molecular characterization of the bacterial isolates to determine sequence type for molecular epidemiology and genotypic mechanism of resistance, and have instead relied only on phenotypic characterization of the mechanism of ceftriaxone resistance. Finally, given study inclusion relied on patient presentation and medical practitioner laboratory referral patterns, we cannot exclude the possibility of inclusion bias.

## Conclusions

These data emphasize the importance of hospitals and RACFs as foci of amplification of CR-EC, and also offer a novel appreciation of the role of sociodemographic factors. While these sociodemographic predictors of CR-EC require further investigation, these data demonstrate the ‘non-random’ nature of antimicrobial resistance in the community. Further understanding of the underlying drivers at play may guide future diagnostic and treatment algorithms to improve patient outcomes and help with strategies to control the dissemination of antimicrobial resistance within the community setting.

## Abbreviations

AGAR: Australian Group on Antimicrobial Resistance
AMR: Antimicrobial resistance
aRR: Adjusted relative risk
BSI: Bloodstream infections
CI: Confidence interval
CLSI: Clinical and Laboratory Standards Institute
CR-EC: Ceftriaxone-resistant *E. coli*
CS-EC: Ceftriaxone-susceptible *E. coli*
*E. coli*: *Escherichia coli*
ED: Emergency department
EDTA: Ethylenediaminetetraacetic acid
ESBL: Extended spectrum ß–lactamases
IQR: interquartile range
IRSD: Index of Relative Socioeconomic Deprivation
MIC: minimum inhibitory concentration
RACF: residential aged care facility
USA: United States of America
VFR: visiting friends and relatives
